# Evaluation of Polygenic Risk Score for Prediction of Childhood Onset and Severity of Asthma

**DOI:** 10.3390/ijms26010103

**Published:** 2024-12-26

**Authors:** Olga Savelieva, Alexandra Karunas, Inga Prokopenko, Zhanna Balkhiyarova, Irina Gilyazova, Irina Khidiyatova, Elza Khusnutdinova

**Affiliations:** 1 Institute of Biochemistry and Genetics, Subdivision of the Ufa Federal Research Centre of the Russian Academy of Sciences, 450054 Ufa, Russia; olyasavelie@yandex.ru (O.S.);; 2 Laboratory of Genomic and Postgenomic Technologies, Federal State Budgetary Educational Institution of Higher Education, Ufa University of Science and Technology, 450076 Ufa, Russia; 3 Faculty of Biology, Federal State Budgetary Educational Institution of Higher Education “Saint-Petersburg State University”, 199034 St. Petersburg, Russia; 4 Department of Medical Genetics and Fundamental Medicine, Federal State Budgetary Educational Institution of Higher Education, Bashkir State Medical University, Russian Ministry of Health, 450008 Ufa, Russia; 5 Department of Clinical & Experimental Medicine, University of Surrey, Guildford GU2 7XH, UK

**Keywords:** asthma, polymorphism, polygenic score, pharmacogenetics, association

## Abstract

Asthma is a common complex disease with susceptibility defined through an interplay of genetic and environmental factors. Responsiveness to asthma treatment varies between individuals and is largely determined by genetic variability. The polygenic score (PGS) approach enables an individual risk of asthma and respective response to drug therapy. PGS models could help to predict the individual risk of asthma using 26 SNPs of drug pathway genes involved in the metabolism of glucocorticosteroids (GCS), and beta-2-agonists, antihistamines, and antileukotriene drugs associated with the response to asthma treatment within GWAS were built. For PGS, summary statistics from the Trans-National Asthma Genetic Consortium GWAS meta-analysis, and genotype data for 882 individuals with asthma/controls from the Volga-Ural region, were used. The study group was comprised of Russian, Tatar, Bashkir, and mixed ethnicity individuals with asthma (*N* = 378) aged 2–18 years. and individuals without features of atopic disease (*N* = 504) aged 4–67 years from the Volga-Ural region. The DNA samples for the study were collected from 2000 to 2021. The drug pathway genes’ PGS revealed a higher odds for childhood asthma risk (*p* = 2.41 × 10^−12^). The receiver operating characteristic (ROC) analysis showed an Area Under the Curve, AUC = 0.63. The AUC of average significance for moderate-to-severe and severe asthma was observed (*p* = 5.7 × 10^−9^, AUC = 0.64). Asthma drug response pathway gene variant PGS models may contribute to the development of modern approaches to optimise asthma diagnostics and treatment.

## 1. Introduction

Asthma is a heterogeneous disease, usually characterized by chronic airway inflammation. There are more than 300 million individuals with asthma worldwide with its prevalences varying between 1% and 29% [1,2]. Asthma occurs through the interaction of internal and external risk factors. Internal factors include genetic predisposition and atopy, while external environmental factors encompass allergens, air pollution, tobacco smoke, infectious diseases, and others. The pathogenesis of asthma is characterized by chronic inflammation of the airways, caused by the activation of various immune cells, such as eosinophils and mast cells. The heritability of asthma is estimated up to 74% in adults and up to 90% in children [3,4]. The effective control of asthma symptoms remains one of the most important treatment goals. The insufficient control of asthma leads to lifestyle limitations with a considerable burden on quality of life, the development of more severe forms, and an increased frequency of disease exacerbations, as well as an increase in the number of individuals with disabilities [5]. Current asthma therapy is pathogenetic and aims to eliminate allergic inflammation of the bronchial mucosa, reduce bronchial hyperreactivity, restore bronchial patency, alleviate bronchospasm, and prevent the structural remodeling of the bronchial wall. The main groups of antiasthmatic drugs include glucocorticosteroids (GCS), beta-2-agonists, antihistamines, and antileukotriene drugs. Inhaled corticosteroids (ICS) are the preferred treatment for asthma in children. In cases where asthma control is inadequate, it is advisable to consider adding a long-acting beta-2 agonist (LABA) to the ICS therapy to enhance management. However, leukotriene modifiers, antihistamines, and, in rare cases, biologics are also used. It is known that the long-term use of ICS can lead to systemic side effects, however, low-to-moderate doses can significantly reduce this risk. The systemic use of LABAs in combination with ICS is considered safe, but their use as a monotherapy should be avoided, as this may increase the risk of serious side effects, including asthma exacerbations [1,2,6,7]. To date, up to 60–80% of variability in the individual response to asthma treatment is influenced by heritable factors [8].

Several hundred genes that contribute significantly to interindividual variability in the antiasthmatic drug response and participate in the asthma pathogenesis have been identified. A number of polymorphic variants capable of influencing the functional activity of genes involved in the development of inflammation, the formation of immune responses, the differentiation of Th2 cells, and the regulation of epithelial barrier function have been identified. These variants are assumed to be one of the possible causes of asthma development and progression. Genes involved in the metabolism of antiasthmatic drugs encode specific receptors regulating inflammation, cell proliferation and differentiation (*NR3C1*, *ADRB2*), and proteins involved in various stages of inflammatory mediator biosynthesis (*HRH1*, *HRH2*, *HRH3*, *HRH4*, *LTC4S*, *ALOX5*, *LTA4*, etc.). A number of studies have shown that polymorphic variants in the above group of genes are associated with the development and course of asthma [9,10,11,12]. For example, asthma drug response involves the gene encoding of the glucocorticoid receptor (*NR3C1*), one of the most studied genes for GCS metabolism. The products of *NR3C1* gene expression are several glucocorticoid receptor isoforms resulting from alternative mRNA splicing (GCRα, GCRβ, GCRδ, GCRγ, and GCR-P) [9]. The rs41423223247*G allele of the *NR3C1* gene was associated with severe asthma in patients from Ukraine [13] and with an increase in the number of asthma exacerbations and severe asthma in children from Egypt [12]. The beta-2-adrenergic receptor ADRB2 is extensively expressed in airway cells. ADRB2 mediates various physiological responses, including bronchodilation, vasodilation, and various anti-inflammatory actions. The rs1042713 and rs1042714 polymorphisms of the *ADRB2* gene were associated with asthma development and severe asthma [11]. The increased expression of the arginase isoenzymes encoded by the *ARG1* and *ARG2* genes leads to the higher production of L-ornithine, polyamines, and L-proline involved in airway remodeling, cell proliferation, and cell fibrosis [14]. The level of the inflammatory mediator histamine is also under genetic control. Proteins encoded by the amino oxidase copper-containing 1 *AOC1* and histamine-N-methyltransferase *HNMT* genes are the key enzymes involved in the first steps of histamine degradation [10]. The activation of histamine receptors HRH1, HRH2, HRH3, and HRH4 by histamine leads to higher cytokine production [15].

Proteins encoded by *LTC4S*, *ALOX5*, and *LTA4* genes are involved in various stages of cysteinyl leukotrienes biosynthesis. Leukotrienes are important lipid mediators exhibiting bronchoconstrictive and proinflammatory activity in asthma [7]. Several GWAS studies of asthma treatment response have also aimed to search for new key links in the disease pathogenesis, which may be associated with the efficacy of certain groups of drugs. To date, more than 20 genome-wide association studies (GWAS) of the response to asthma treatment showed a large number of loci associated with individual sensitivity to asthma treatment, including variants at *GLCCI1*, *TBXT*, *FBLX7*, *ALLC*, *SPATS2L*, *THRB*, *SLC22A15*, *ALDH7A1*, *PSAP*, and *SCG3* (http://www.ebi.ac.uk/gwas/, accessed on 10 September 2024). For a number of genes associated with susceptibility to asthma treatment by GWAS, data revealed a role in the predisposition to asthma development. The *FBXL7* gene was suggested to be involved in the pathogenesis of airway inflammation in asthma as a factor contributing to the degradation of cytokine receptors or degradation of the subunit factor induced by hypoxia [16]. The expression of the *GLCCI1* gene was detected in lung tissue and in immune cells and the association of rs11976862*GG genotype of the *GLCCI1* gene with asthma in Chinese asthma patients using ICS were revealed [17,18].

An actively used method for the individual prediction of genetic predisposition to heritable characteristics is the polygenic score (PGS). PGS modeling is used to assess the risk of a disease and quantitative phenotypes, to develop clinically applicable genetic predictions for complex traits and diseases, and to compare the predictive ability of such scores between populations and various ethnic groups [19]. An important problem for modern genetic studies is the portability of the constructed PGS for individuals from another population, associated with a number of factors [20]. One of the factors is the genetic structure features of the population, including differences in the frequency of risk alleles, patterns of linkage disequilibrium, and the strength of the trait effect [21,22]. On the other hand, environmental factors such as age, sex, and social and economic status also influence the heritability of traits [23]. The above differences can significantly affect the accuracy of predicting the risk of disease development, even among individuals of the same origin [24]. In particular, several asthma studies confirmed the existence of differences in analyzing the same PGS model among individuals from different backgrounds [25,26,27,28]. To date, most PGS models for asthma were developed for Europeans. However, no PGS models for asthma specific to individuals from Volga-Ural region (VUR) of Russia were published. Thus, due to the fact that the functional activity of genes involved in the metabolism of antiasthmatic drugs was significantly associated with the known key links of asthma pathogenesis, and certain polymorphisms of these genes were associated with asthma development according to the published data and previous results, it is important to study the role of polymorphic variants of antiasthmatic drug genes in view of their participation in asthma development. PGS models were defined to evaluate individual risk of asthma based on 26 SNPs at genes involved in the metabolism of major groups of asthma medications or associated with the response to asthma treatment using GWAS data in individuals with a childhood onset asthma from VUR.

## 2. Results

### 2.1. Association Analysis

Twenty-six SNPs located in genes involved in the metabolism of antiasthmatic drugs or the report to asthma treatment were dissected based on GWAS with the asthma in case/control groups from VUR by analysing these variants using a logistic regression approach. The strongest association of rs295137 located near the *SPATS2L* gene (β = 0.4707, SE = 0.1323, *p* = 0.0004) and the rs2395672 in the intron of the *CMTR1* gene with a childhood onset asthma (β = 0.4433, SE = 0.129, *p* = 0.0006) were established (Table S1). The association of the rs2395672*G allele of the *CMTR1* gene (β = 0.6816, SE = 0. 0.1838, *p* = 0.0002) with moderate-to-severe and severe asthma was also established (Table S2).

Association analysis of the weighted PGS for asthma using β coefficients from logistic regression as the weights revealed higher risk of childhood onset asthma in cases compared controls (OR [95% CI] = 1.67 [1.45; 1.94]; *p* = 2.41 × 10^−12^) (Table 1). The analysis of weighted polygenic risk models for moderate-to-severe and severe asthma (OR [95% CI] = 1.8 [1.48; 2.21]; *p* = 5.7 × 10^−9^) also found significant differences in individuals with asthma compared to controls.

### 2.2. ROC Analysis

**Figure 1:**
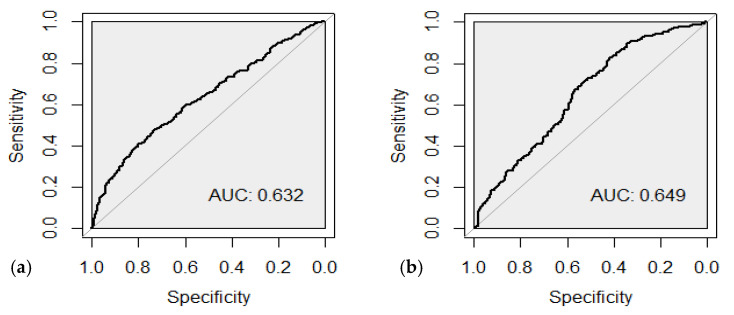
ROC curve evaluating the discriminatory ability of the weighted PGS model for the assessment of childhood onset asthma (**a**) and moderate-to-severe and severe asthma (**b**).

The ROC analysis of the constructed PGS models was performed to compare their prognostic power for estimating the risk of childhood onset asthma and severe asthma. The area under the ROC curve in the weighted PGS predicted the risk of developing childhood onset asthma with an AUC of 0.632 (95% CI [0.60; 0.67]), which indicates an “average” predictive ability of the model to identify individuals with asthma with a high risk of this disease. The sensitivity of the weighted PGS for asthma was 0.48, and the specificity of 0.74 (Figure 1a). The area under the AUC curve according to the ROC analysis of the weighted PGS predicting the risk of moderate-to-severe and severe asthma course was 0.649 (95% CI [0.60; 0.69]), with the sensitivity of 0.41 and specificity of 0.84 (Figure 1b).

## 3. Discussion

Asthma is a widespread disease throughout the world, with an increasing incidence. The hypodiagnosis and underdiagnosis of asthma remains one of the most important problems of modern medicine. The inflammatory process determines the risk of exacerbations, cumulative pathological changes in respiratory function, and various structural changes in the pathogenesis of asthma [6]. Several studies have suggested that genes involved in the metabolism of antiasthmatic drugs play an important role in the development of inflammation and predisposition to asthma development [8,10,11,12,13,14]. In this paper, the prognostic ability of gene polymorphisms involved in the metabolism of drugs used for pathogenetic asthma treatment or associated with the response to asthma medications according to GWAS was evaluated to determine the risk of asthma development and severe disease course in individuals from the Volga-Ural region. The combination of the 26 thoroughly selected SNPs was found to predict a higher chance of asthma development and severe course. Note that the study included a group of individuals with an exact clinical diagnosis of asthma made by qualified specialists on the basis of clinical, general laboratory, and additional methods of examination in accordance with the criteria of program documents on the diagnosis, treatment, and prevention of this disease. This is important due to the fact that some studies of asthma use groups of individuals with a diagnosis of asthma made on the basis of cohort and cross-sectional population surveys without its confirmation by a specialist.

Most inheritance of common multifactorial diseases, such as asthma, is now thought to be mediated by multiple genetic variants, with each making a limited contribution to trait formation. A significant proportion of polymorphic loci were identified in GWAS studies [19,29]. The use of a polygenic risk score approach allows the results of several genetic studies to be combined and to make an evaluation of the genetic predisposition to a trait at the individual level [30]. This could become a tool for using the constructed PGS models in a clinical practice later.

The values of the prognostic significance level of the AUC curves for the constructed PGS models of asthma found in the current study is compatible with the results of earlier studies. Spycher and colleagues constructed PGS models of asthma within Europeans based on polymorphic variants of genes previously associated with asthma development in children according to the GABRIEL Consortium data, which showed a low ability of the models to predict different asthma phenotypes and wheezing (AUC < 0.60). This study confirmed not only that SNPs attained genome-wide significance in GWAS, but also SNPs with lower scores contribute to asthma prediction [25]. Sordillo J.E. et al. also obtained asthma PGS based on 41 genome-wide significant SNPs from TAGC GWAS [31] for Non-Hispanic Whites, Asians, Hispanics, and Blacks with low prognostic ability for disease prediction (AUC < 0.60). The authors note that it is important to select GWAS studies underlying PGS specifically for groups of individuals of different ancestry because variants associated with pathology may be missed if they are absent or have low frequency in Europeans [27]. Belsky D.W. and others constructed a PGS model for life-course-persistent asthma in individuals with childhood onset asthma with average prognostic significance based on 15 genes polymorphisms that have reached genome-wide significance in a GWAS study of 1037 individuals from New Zealand (AUC = 0.61). It was found that childhood-onset asthma cases at higher genetic risk were more likely to develop life-course-persistent asthma, atopy, airway hyperresponsiveness, and incompletely reversible airway obstruction than cases at lower genetic risk [26]. Kothalawala D.M. and colleagues constructed a PGS model of asthma development based on 105 gene polymorphisms associated with early asthma manifestation in individuals of European origin having average predictive value (AUC = 0.61) [28].

Several studies analyzed the interactions between environmental risk factors and genetic variation in susceptibility to asthma. Zhu Y. et al. developed a PGS based on 212 loci identified in GWAS studies of participants with European ancestry. A higher incidence of asthma correlated with an increase in genetic risk was found. The size of the effect of air pollution was greater among subgroups with low genetic risk or unfavorable lifestyles [32]. Liang H. et al. categorised the risk of incident asthma as three groups based on the tertiles of PGS based on 212 associated with asthma in Europeans. Compared to ideal lifestyle in a low genetic risk group, poor lifestyle was associated with a hazard ratio of up to 3.87 for developing asthma in a high genetic risk group. A healthy lifestyle score was constructed using body mass index, smoking status, physical activities, and dietary pattern [33].

The systematic assessment of polygenic risk scores for various polygenic traits was carried out in a number of studies. Mars N. et al. explored the dynamic for three types of family history across 24 common diseases including asthma. The constructed PGSs explained on average one-tenth of the family history of first-degree relatedness. PGS based on 668,286 SNPs associated with asthma in individuals of European ancestry identified a 1.43 times higher risk of asthma in Europeans [34]. Tanigawa Y. et al. conducted a systematic assessment of PGS prediction across more than 1500 traits using data from the UK Biobank. A set of asthma PGSs contains from 435 to 8508 SNPs associated with asthma in Europeans according to UK Biobank data were developed. AUC values for asthma PGS ranged from 0.57 to 0.67 [35]. Privé F. et al. investigated the extent to which PGSs are transferable between ancestries by deriving polygenic scores for 245 curated traits from the UK Biobank data and applying them in nine ancestry groups. Averaged over all studied phenotypes including asthma, compared to the prediction performance in individuals of Northwestern European ancestry, the relative predictive ability in terms of partial-r2 was 18% for Africans, 49% for East Asians, 65% for South Asians, and 94% for Northeast Europeans [36].

The average values of the constructed PGS models of asthma’s prognostic ability level based on different gene polymorphisms confirm the difficult complex nature and phenotypic heterogeneity of this disease, which is formed by the interaction of hereditary predisposition, environmental factors, and epigenetic changes.

A limitation of this study is the relatively small sample size, the defined selection of SNPs, and the absence of clinical predictors in the model. However, the main aim of the study was to develop a PGS specifically for individuals from the Volga-Ural region, for which both the results of the GWAS study and genotyping data from an independent sample of individuals were available. Additionally, the study aimed to understand the potential relevance of complex analyses of the variants included in this model for assessing the individual risk of disease development. The observed results should be validated in a larger sample of individuals.

## 4. Materials and Methods

### 4.1. Study Group

The study group included 882 unrelated individuals with and without asthma, consisting of individuals without features of atopic diseases from the VUR. The study participants were matched for gender, age, and ethnicity. The asthma group consisted of 378 individuals aged 2–18 years (265 male, 113 female) comprising several ethnicities: 94 Russians, 111 Tatars, 53 Bashkirs, and 120 individuals of mixed ethnicity. The inclusion criteria for patients in the study were a diagnosis of asthma determined by pulmonologists based on a clinical examination, family and medication history, and general laboratory and additional methods of investigation in accordance with the criteria GINA and National Program documents on the diagnosis, treatment, and prevention of diseases [1,6]. In addition, individuals with asthma included in the study were receiving ICS or combination therapy (ICS-long-acting beta-2-agonist) at a daily dose of 100 to 1000 µg fluticasone propionate, depending on the severity of the course and period of the disease, for at least 3 months. In contrast, the absence of a clinically confirmed diagnosis of asthma, as well as the presence of other respiratory diseases such as chronic obstructive pulmonary disease, tuberculosis, or other active respiratory infections, were the criteria for excluding patients from the study. All participants with asthma were from the Children’s Department of the Bashkir State Medical University Hospital, the Pulmonology and Allergology Departments of the Municipal Clinical Hospital number 21, Ufa city, and the Allergology Department of the Republican Children’s Clinical Hospital, Ufa city. Individuals without asthma, or bronchopulmonary, allergic, and autoimmune diseases, or allergic heredity, included 504 participants aged 4 to 67 years (213 male, 291 female) comprising 194 individuals of Russian, 145 of Tatar, 86 of Bashkir ethnicity, and 79 individuals of mixed ethnicity, and these were defined as controls. The criteria for including individuals in the control group were the absence of asthma, bronchopulmonary, allergic and autoimmune diseases, and allergic heredity. Exclusion criteria for controls were serum total immunoglobulin E (IgE) values exceeding 100 IU/mL and the presence of abnormalities in spirometry or picofluorometry. The ethnic origin of the participants was determined on the basis of information about their ethnicity in both parental lines in three generations. All individuals with asthma over the age of 15 gave informed consent to participate in the study. Children under the age of 15 with asthma were enrolled in the study with the consent of their parents.

### 4.2. DNA Extraction, Genotyping

The selection of 26 SNP variants for genotyping was based on published data demonstrating the involvement of protein products of genes in the metabolism of drugs for asthma treatment, as well as on GWAS data on the response to asthma medications [10,11,14,15,16,37,38,39,40,41,42,43,44] (Table S3). All SNPs had minor allele frequency (MAF) > 0.01 in Europeans. DNA was isolated from peripheral blood by phenol-chloroform extraction [45]. Blood was collected in tubes with K3 EDTA suppliant. It was thoroughly mixed and stored at 4 °C, then DNA extraction was carried out. DNA concentration was measured by a NanoDrop 1000 spectrophotometer (Thermo Fisher Scientific, Fitchburg, WI, USA). Genomic DNA samples were normalized at 50 ng/μL. The genotyping of 26 SNPs was performed using real-time PCR (TestGen LLC, DNA-Synthesis, Moscow, Russia) and competitive allele-specific PCR (KASP, Maxim Medical LLC, Moscow, Russia) in accordance with the protocols of the company manufacturer using a CFX96 Real-Time PCR Detection System (Bio-Rad, Hercules, CA, USA) and a QuantStudio 12K Flex AccuFill system (Applied Biosystems, Foster City, CA, USA).

### 4.3. Quality Control, Association Analysis

The selection of 26 SNPs for PGS analysis excluded polymorphisms in linkage disequilibrium (r2 > 0.2). Two datasets were used in the study—summary statistics of the Trans-National Asthma Genetic Consortium (TAGC) meta-analysis of GWAS [31] and genotype data for cases and controls from the Volga-Ural region populations. No significant deviation from the Hardy–Weinberg equilibrium in each of the SNPs was found (*p* > 0.05). The logistic regression analysis of studied SNPs with asthma using a log-additive genetic model was performed to calculate the effect sizes (β coefficient). Calculations were performed applying PLINK 1.9 [46] and the software tool and environment R 4.1.1 [47]. The SNP variants selected for the PGS analysis are shown in the Table 2.

The TAG Consortium (TAGC) conducted multiancestry meta-analysis of worldwide asthma GWAS (23,948 asthma cases, 118,538 controls) of individuals from ethnically diverse populations: European, African, Japanese, and Latino ancestries [31]. The TAGC meta-analysis of GWAS included data from GWAS conducted earlier on the cohort of individuals with asthma and controls from the VUR (UFA cohort) as part of the GABRIEL Project (EC Framework 6 grant) [48,49,50], and the European Genome-phenome Archive (EGA)—https://ega-archive.org/datasets/EGAD00000000103 (accessed on 10 September 2024) and https://ega-archive.org/datasets/EGAD00000000104 (accessed on 10 September 2024) consisting of a small proportion of individuals with asthma/controls overlapping with the sample from this paper. Due to this fact, the correction of TAGC GWAS results was performed by recalculating SNP effect size values using R 4.1.1 software based on an inverse version of the formula for meta-analysis with fixed inverse variance effects [51].

### 4.4. The Calculation of Polygenic Risk Score

The PGS was calculated for each individual as the risk allele effect weighted is the sum of all the studied SNPs. The β coefficient values were computed by logistic regression analysis performed on the study sample of individuals with asthma and controls or adjusted β coefficient values from the TAGC meta-analysis of GWAS studies [31] were used as weights for the polygenic risk analysis. The risk alleles for PGS were chosen based on the results of the logistic regression analysis of asthma in the combined case/control group from the Volga-Ural region. For a number of polymorphisms for which TAGC GWAS summary statistics data were not available (rs13182402, rs41423247, rs2781667, rs37973, rs1049793, rs7140310, rs11000016), β-coefficient values of SNPs with strong linkage disequilibrium (r2 > 0.08, rs6861395, rs853180, rs2608897, rs37972, rs11978239, rs17140310, rs11000000, respectively) were used as weights for the PGS analysis. Linkage disequilibium between SNPs was defined based on the European and Asian populations LD estimates from the 1000 Genomes Project (https://ldlink.nci.nih.gov/, accessed on 10 September 2024).

### 4.5. Analysis of ROC Curves

The distributions of the individual PGSs were compared between the individuals with asthma and without asthma (controls) using ROC curves in the R 4.1.1 environment with the pROC package [52]. The prognostic power of the models for detecting the risk of asthma was estimated by comparing the AUC values of the relevant ROC curves, which compares the frequency of true-positive and false-positive rates.

## 5. Conclusions

In this study, a number of PGS models were constructed based on drug pathway gene polymorphisms associated with the childhood onset and severity of asthma with average prognostic significance in this study. Further studies comprising larger samples and additional genetic, clinical, and environmental parameters may enhance the proposed model and improve its predictive power. PGS should be validated in independent groups before implementation. Additional prospective studies may also be useful to assess the clinical utility of PGS in practical settings. The prospect of implementing PGS in clinical practice opens new frontiers for identifying groups at high risk of developing the disease, which, in turn, will enable timely and targeted medical care for them.

## Figures and Tables

**Table 1:** The results of the PGS analysis of asthma using 26 SNPs of genes involved in the metabolism of drugs used for asthma treatment or associated with the report on asthma medications according to GWAS.

Group	Weighted Analysis Using β-Coefficients Obtained in the Group from VUR		Weighted Analysis Using Corrected GWAS β-Coefficients		N_A_	N_C_
	*p*	OR [95% CI]	*p*	OR [95% CI]		
Individuals with childhood onset of asthma—controls	2.41 × 10^−12^	1.67 [1.45; 1.94]	0.64	1.03 [0.90; 1.18]	378	542
Individuals with moderate-to-severe and severe asthma—controls	5.7 × 10^−9^	1.8 [1.48; 2.21]	0.35	0.92 [0.78; 1.09]	174	542

Legend: N_A_-number of individuals with asthma, N_C_-number of individuals from the control group.

**Table 2:** General characteristics of SNPs.

Gene	Chromosome	Position	SNP ID	Rare Allele/Alternate Allele	MAF in the Control Group, %	MAF in Europeans (CEU, 1000 Genomes), %
*SLC22A15*	1	116,411,750	rs1281744	C/T	11.27	13.10
*ALLC*	2	3,700,901	rs11123610	C/T	37.83	34.80
*SPATS2L*	2	200,858,285	rs295137	T/C	34.31	41.90
*HRH1*	3	11,275,707	rs901865	A/G	15.26	20.20
*THRB*	3	24,513,842	rs892940	T/C	43.93	41.90
*FBXL7*	5	15,836,596	rs10044254	C/T	26.15	18.70
*ALDH7A1*	5	125,918,148	rs13182402	G/A	10.35	10.60
*NR3C1*	5	142,778,575	rs41423247	G/C	35.04	64.10
*ADRB2*	5	148,206,440	rs1042713	A/G	40.93	34.80
*HRH2*	5	175,041,825	rs2067474	A/G	3.81	3.00
*LTC4S*	5	179,153,244	rs730012	C/A	24.49	32.30
*CMTR1*	6	37,428,577	rs2395672	A/G	20.71	18.20
*ARG1*	6	131,895,144	rs2781667	T/C	33.16	28.80
*TFT*	6	166,499,260	rs2305089	T/C	45.32	59.10
*GLCCI1*	7	800,787	rs37973	C/T	46.73	47.50
*CRHR2*	7	30,709,475	rs2190242	C/A	35.70	18.70
*MAGI2*	7	77,641,211	rs2691529	C/T	26.91	21.70
*AOC1*	7	150,557,665	rs1049793	G/C	34.77	29.30
*ALOX5*	10	45,221,095	rs2115819	C/T	47.96	40.90
*PSAP*	10	73,579,217	rs11000016	T/C	15.92	19.70
*LTA4*	12	94,962,684	rs2660845	G/A	28.81	29.30
*ARG2*	14	6,811,073	rs7140310	C/A	15.16	10.60
*ARG2*	14	67,184,754	rs3742879	G/A	29.67	33.80
*SCG3*	15	49,756,960	rs17525472	C/T	12.92	12.30
*ADCY9*	16	3,973,437	rs2230739	C/T	34.60	37.90
*HRH4*	18	20,310,764	rs11665084	T/C	12.20	9.60

## Data Availability

The additional data are available in the Supplementary Materials.

## Supplementary Materials

The following supporting information can be downloaded at: https://www.mdpi.com/article/10.3390/ijms26010103/s1.
