# Supplementary material for: Evaluation of Polygenic Risk Score for Prediction of Childhood Onset and Severity of Asthma
Source: Int J Mol Sci. 2024 Dec 26;26(1):103. doi: 10.3390/ijms26010103 (PMC11719589; doi:10.3390/ijms26010103)
Supplement: Supplementary file 1 [file ijms-26-00103-s001.zip › ijms-3343501-supplementary.pdf]

Table S1. Results of the regression analysis of the association of the studied SNPs with child onset asthma

№	Gene	SNP	Alleles A1/A2	A1 allele frequency in patients,%	A1 allele frequency in controls,%	β±SE	p
1	*SLC22A15*	rs1281744	C/T	12.10	11.27	0.0807±0.1761	0.6469
2	*ALLC*	rs11123610	T/C	63.19	62.17	0.0446±0.1063	0.6745
3	*SPATS2L*, chr2: 200285317	rs295137	C/T	75.70	65.69	0.4707±0.1323	0.0004
4	*HRH1*	rs901865	A/G	18.73	15.26	0.2285±0.1464	0.1187
5	*THRB*	rs892940	C/T	56.37	56.07	0.0112±0.1169	0.9235
6	*FBXL7*	rs10044254	T/C	79.67	73.85	0.3081±0.1203	0.0104
7	*ALDH7A1*	rs13182402	G/A	11.20	10.35	0.0874±0.1527	0.5670
8	*NR3C1*	rs41423247	G/C	35.64	35.04	0.0247±0.0964	0.7975
9	*ADRB2*	rs1042713	G/A	62.47	59.07	0.1392±0.0976	0.1541
10	*HRH2*	rs2067474	A/G	4.26	3.81	0.1102±0.2357	0.6401
11	*LTC4S*	rs730012	A/C	76.27	75.51	0.0437±0.1146	0.7031
12	*CMTR1*	rs2395672	G/A	85.70	79.29	0.4433±0.129	0.0006
13	*ARG1*	rs2781667	C/T	70.33	66.84	0.1596±0.1104	0.1484
14	*TFT*	rs2305089	T/C	46.13	45.32	0.0324±0.1016	0.7501
15	*GLCCI1*	rs37973	C/T	49.86	46.73	0.1252±0.1038	0.2278
16	*CRHR2*	rs2190242	C/A	36.54	35.70	0.0338±0.0989	0.7325
17	*MAGI2*	rs2691529	C/T	31.95	26.91	0.2587±0.1080	0.0166
18	*AOC1*	rs1049793	C/G	68.05	65.23	0.1229±0.0999	0.2186
19	*ALOX5*	rs2115819	T/C	53.61	52.04	0.0672±0.0986	0.4952
20	*PSAP*	rs11000016	C/T	86.27	84.08	0.1741±0.1356	0.1989
21	*LTA4*	rs2660845	A/G	71.91	71.19	0.0371±0.1084	0.7321
22	*ARG2*	rs7140310	C/A	16.76	15.16	0.1262±0.1587	0.4265
23	*ARG2*	rs3742879	G/A	29.89	29.67	0.0105±0.1027	0.9185
24	*SCG3*, chr15:51677 471	rs17525472	C/T	12.93	12.92	0.0014±0.142	0.9924
25	*ADCY9*	rs2230739	T/C	66.46	65.40	0.0442±0.1236	0.7207
26	*HRH4*	rs11665084	C/T	89.71	87.80	0.2017±0.1564	0.1972

Legend: A1- risk allele in the merged group from VUR, A2-alternative allele

Table S2. Results of the regression analysis of the association of the studied SNPs with moderate-to-severe and severe asthma in children

№	Gene	SNP	Alleles A1/A2	A1 allele frequency in patients,%	A1 allele frequency in controls,%	β±SE	p
1	*SLC22A15*	rs1281744	C/T	12.66	11.27	0.1328±0.217	0.5407
2	*ALLC*	rs11123610	T/C	61.35	62.17	-0.0365±0.1379	0.7913
3	*SPATS2L*, chr2: 200285317	rs295137	C/T	71.07	65.69	0.2459±0.1626	0.1305
4	*HRH1*	rs901865	A/G	18.71	15.26	0.2229±0.1781	0.2106
5	*THRB*	rs892940	C/T	57.50	56.07	0.0530±0.1448	0.7145
6	*FBXL7*	rs10044254	T/C	79.45	73.85	0.2883±0.1534	0.0602
7	*ALDH7A1*	rs13182402	G/A	11.27	10.35	0.0923±0.1943	0.6349
8	*NR3C1*	rs41423247	G/C	31.21	35.04	-0.1598±0.1276	0.2104
9	*ADRB2*	rs1042713	G/A	61.56	59.07	0.1006±0.1256	0.4233
10	*HRH2*	rs2067474	A/G	4.91	3.81	0.2580±0.2910	0.3754
11	*LTC4S*	rs730012	A/C	76.16	75.51	0.0375±0.1491	0.8014
12	*CMTR1*	rs2395672	G/A	88.66	79.29	0.6816±0.1838	0.0002
13	*ARG1*	rs2781667	C/T	70.06	66.84	0.1459±0.1413	0.3020
14	*TFT*	rs2305089	T/C	44.24	45.32	-0.0409±0.1265	0.7466
15	*GLCCI1*	rs37973	C/T	49.09	46.73	0.0911±0.1298	0.4828
16	*CRHR2*	rs2190242	C/A	36.88	35.70	0.0470±0.1286	0.7150
17	*MAGI2*	rs2691529	C/T	32.27	26.91	0.2689±0.1377	0.0508
18	*AOC1*	rs1049793	C/G	69.94	65.23	0.2104±0.1324	0.1121
19	*ALOX5*	rs2115819	T/C	51.46	52.04	-0.0240±0.1264	0.8493
20	*PSAP*	rs11000016	C/T	85.55	84.08	0.1141±0.1745	0.5130
21	*LTA4*	rs2660845	A/G	70.00	71.19	-0.0611±0.1406	0.6640
22	*ARG2*	rs7140310	C/A	15.26	15.16	0.0083±0.2005	0.9670
23	*ARG2*	rs3742879	G/A	33.72	29.67	0.1877±0.1323	0.1561
24	*SCG3*, chr15:51677471	rs17525472	C/T	10.98	12.92	-0.1916±0.1982	0.3338
25	*ADCY9*	rs2230739	T/C	65.25	65.40	-0.0064±0.1548	0.9667
26	*HRH4*	rs11665084	C/T	89.60	87.80	0.1939±0.207	0.3488

Table S3. Gene and Protein Characteristics

Gene	Official Full Name	Localisation	Protein function
SLC22A15	Solute carrier family 22 member 15	1p13.1	SLC22A15 is an organic ion transporter that carries a variety of medically and physiologically important compounds, including drugs, toxins, hormones, neurotransmitters, and cellular metabolites (https://www.ncbi.nlm.nih.gov/gene/55356). A high level of *SLC22A15* gene expression was detected in lung and bronchial epithelial cells. Polymorphisms of the SLC22A15 gene were associated with the sensitivity of asthma patients to the use of beta-2 agonists [37].
ALLC	Allantoicase	2p25.3	Allantoinase belongs to the class of hydrolases that catalyze the hydrolysis of non-peptide C-N bonds and is involved in purine metabolism. In GWAS, a number of polymorphic variants of the *ALLC* gene were associated with changes in Forced Expiratory Volume in 1 second (FEV1) in patients undergoing GCS treatment. It is known that uric acid levels in patients with allergic asthma who are not receiving corticosteroid therapy are significantly higher than in those receiving this treatment, which may indicate a role for the ALLC in the metabolism of GCS [38]."
SPATS2L	Spermatogenesis associated serine rich 2 like	2q33.1	SPATS2L enables RNA binding activity. MicroRNA-mediated knockdown of the *SPATS2L* gene increases the expression level of the *ADRB2* gene and the efficacy of beta-2-agonist treatment [39].
HRH1	Histamine receptor H1	3p25.3	The HRH1 protein mediates the contraction of smooth muscles, increases capillary permeability due to the contraction of terminal venules, promotes the release of catecholamines from the adrenal medulla, and facilitates neurotransmission in the central nervous system. The effects of the inflammatory mediator histamine are realized through its interaction with histamine receptors, including HRH1. The interaction of histamine with HRH1, HRH2, HRH3, and HRH4 receptors leads to their activation in the organism and increased production of cytokines [10; 15].
THRB	Thyroid hormone receptor beta	3p24.2	The thyroid hormone receptor is located in the nucleus and, after binding to thyroid hormone, regulates (both suppresses and activates) transcription by binding to retinoid X receptor beta (RXRB). Thyroid hormone is involved in the growth and development of the lungs as well as other organs during prenatal and postnatal stages. An association of rs892940 of the *THRB* gene with the sensitivity of asthma patients to the use of beta-2-agonists was found [40].
FBXL7	F-box and leucine rich repeat protein 7	5p15.1	FBXL7 is a member of the F-box protein family, which is characterized by a 42-48 amino acid motif called the F-box, that binds to the S-phase kinase-

			associated protein 1 (Skp1). F-box proteins constitute one of the four subunits of E3 ubiquitin protein ligases known as SCFs (SKP1-Cul1-F-box), which play a role in phosphorylation-dependent ubiquitination of proteins. FBXL7 is thought to be involved in the pathogenesis of airway inflammation in asthma as a factor contributing to cytokine receptor degradation or degradation of the hypoxia-inducible factor (HIF) subunit, which is negatively regulated by FBW7-mediated degradation in hypoxia [16; 41].
ALDH7A1	Aldehyde dehydrogenase 7 family member A1	5q23.2	ALDH7A1 is a member of subfamily 7 in the aldehyde dehydrogenase gene family. These enzymes are thought to play a major role in the detoxification of aldehydes and lysine catabolism (https://www.ncbi.nlm.nih.gov/gene/23194). ALDH7A1 is involved in the degradation of histamine, which is one of the major mediators of inflammation [10].
NR3C1	Nuclear receptor subfamily 3 group C member 1	5q31.3	The glucocorticoid receptor protein can function both as a transcription factor that binds to glucocorticoid response elements in the promoters of glucocorticoid responsive genes to activate their transcription and as a regulator of other transcription factors. It is involved in inflammatory responses, cellular proliferation, and differentiation in target tissues (https://www.ncbi.nlm.nih.gov/gene/23194)
ADRB2	Adrenoceptor beta 2	5q32	The β2-adrenergic receptor is member of the G protein-coupled receptor family and is prominently expressed in the respiratory tract. ADRB2 plays a crucial role in mediating several physiological responses in the airways, such as bronchodilation, vasodilation, and the enhancement of mucociliary clearance. Additionally, it contributes to various anti-inflammatory mechanisms, including the stabilization of mast cells, the regulation of cytokine production, and the reduction of protein leakage in the airways. Therefore, inhaled β2-AR agonists are commonly employed as the first-line bronchodilators for the management of asthma [11].
HRH2	Histamine receptor H2	5q35.2	Histamine receptor H2 belongs to the family 1 of G protein-coupled receptors. It is an integral membrane protein and regulates gastrointestinal motility, cell growth and differentiation (https://www.ncbi.nlm.nih.gov/gene/3274). The effects of the inflammatory mediator histamine are realized through its interaction with histamine receptors, including HRH2. The interaction of histamine with HRH1, HRH2, HRH3, and HRH4 receptors leads to their activation in the organism and increased production of cytokines [10; 15].
LTC4S	Leukotriene C4 synthase	5q35.3	LTC4S protein is an enzyme that catalyzes the first step in the biosynthesis of cysteinyl leukotrienes,

			which are potent biological compounds derived from arachidonic acid. Leukotrienes have been implicated as mediators of anaphylaxis and inflammatory conditions, such as human bronchial asthma (https://www.ncbi.nlm.nih.gov/gene/4056)
CMTR1	Cap methyltransferase 1	6p21.2	Enables mRNA (nucleoside-2'-O-)-methyltransferase activity and is involved in 7-methylguanosine mRNA capping (https://www.ncbi.nlm.nih.gov/gene/23070). The CMTR1 protein is also involved in intracellular defense mechanisms against viral infections. A GWAS of individuals of European descent showed that the rs2395672 polymorphism of the CMTR1 gene is associated with an increased frequency of asthma exacerbations in patients on ICS treatment, suggesting a role for the CMTR1 protein in GCS metabolism [42].
ARG1	Arginase 1	6q23.2	Arginase is an enzyme that catalyzes the hydrolysis of L-arginine to ornithine and urea. Two isoenzymes, arginase type I and II, are encoded by the genes ARG1 and ARG2. Overproduction of Th2 cytokines (IL-4, IL-13) and TGF-β in asthma leads to increased expression of arginase and consequently an increased production of L-ornithine, polyamines, and L-proline, which are involved in processes such as airway remodeling, cell proliferation, increased collagen production, and cell fibrosis [14].
TFT	T-box transcription factor T	6q27	The TFT protein is an embryonic nuclear transcription factor that binds to a specific DNA element known as the palindromic T-site. It binds through a region in its N-terminus called the T-box and affects the transcription of genes required for mesoderm formation and differentiation, including lung development (https://www.ncbi.nlm.nih.gov/gene/6862). The interaction of TBXT with endogenous GCS during fetal and neonatal lung development may further determine the sensitivity of individuals to glucocorticoid usage [43].
GLCCI1	Glucocorticoid induced 1	7p21.3	The function of the GLCCI1 protein is not sufficiently explored. The expression of the *GLCCI1* gene is induced by glucocorticoids and may serve as an early marker for glucocorticoid-induced apoptosis (https://www.ncbi.nlm.nih.gov/gene/113263).<br>GWAS revealed that the polymorphisms rs37972 and rs37973, located in the promoter region of the *GLCCI1* gene, were associated with a reduced response to glucocorticoid treatment in asthma patients. It was found that these polymorphisms affect the level of *GLCCI1* gene transcription [44].
CRHR2	Corticotropin releasing	7p14.3	CRHR2 belongs to the G-protein coupled receptor 2 family, and the subfamily of corticotropin releasing hormone receptor. This receptor shows high affinity

	hormone receptor 2		for corticotropin releasing hormone (CRH) and plays an important role in coordinating the endocrine, autonomic, and behavioral responses to stress and immune challenge (https://www.ncbi.nlm.nih.gov/gene/1395).
MAGI2	Membrane associated guanylate kinase, WW and PDZ domain containing 2	7q21.11	MAGI2 belongs to a family of membrane-associated guanylate kinases containing a core domain that is required for mitotic spindle alignment along the cortical polarity axis. A GWAS of individuals of European origin revealed a tendency for association of the rs2691529 polymorphism of the *MAGI2* gene with the frequency of asthma exacerbations in individuals on ICS treatment. It is assumed that MAGI2 plays a role in the metabolism of glucocorticoids [42]."
AOC1	Amine oxidase copper containing 1	7q36.1	AOC1 is a metal-binding membrane glycoprotein that catalyzes the degradation of histamine through oxidative deamination, as well as the breakdown of compounds involved in allergic and immune responses, cell proliferation, tissue differentiation, and apoptosis [10].
ALOX5	Arachidonate 5-lipoxygenase	10q11.21	ALOX5 is a member of the lipoxygenase gene family and plays a dual role in the synthesis of leukotrienes from arachidonic acid. Leukotrienes are important mediators of a number of inflammatory and allergic conditions. Mutations in the promoter region of *ALOX5* gene lead to a diminished response to antileukotriene drugs used in the treatment of asthma (https://www.ncbi.nlm.nih.gov/gene/240)
PSAP	Prosaposin	10q22.1	PSAP behaves as a myelinotrophic and neurotrophic factor, these effects are mediated by its G-protein-coupled receptors, GPR37 and GPR37L1, undergoing ligand-mediated internalization followed by ERK phosphorylation signaling (https://www.uniprot.org/uniprotkb/P07602/entry). It is involved in the metabolism of histamine [10].
LTA4	Leukotriene A-4 hydrolase	12q23.1	Bifunctional zinc metalloenzyme with both epoxide hydrolase (EH) and aminopeptidase activities. It acts as an epoxide hydrolase to catalyze the conversion of LTA4 into the pro-inflammatory mediator leukotriene B4 (LTB4). In addition to its pro-inflammatory EH activity, it may also mitigate inflammation through its aminopeptidase activity, which inactivates the tripeptide Pro-Gly-Pro (PGP) by cleavage. Furthermore, it is involved in the biosynthesis of resolvin E1 and 18S-resolvin E1 from eicosapentaenoic acid, two lipid mediators that exhibit strong anti-inflammatory and pro-resolving effects." (https://www.uniprot.org/uniprotkb/P09960/entry).
ARG2	Arginase 2	14q24.1	Arginase is an enzyme that catalyzes the hydrolysis of L-arginine to ornithine and urea. Two isoenzymes, arginase type I and II, are encoded by the genes

			ARG1 and ARG2. Overproduction of Th2 cytokines (IL-4, IL-13) and TGF-β in asthma leads to increased expression of arginase and consequently an increased production of L-ornithine, polyamines, and L-proline, which are involved in processes such as airway remodeling, cell proliferation, increased collagen production, and cell fibrosis [14].
SCG3	Secretogranin III	15q21.2	The protein SCG3 is a member of the chromogranin/secretogranin family of neuroendocrine secretory proteins. Granins may serve as precursors for biologically active peptides. Some granins have been shown to function as helper proteins in sorting and proteolytic processing of prohormones (https://www.ncbi.nlm.nih.gov/gene/29106). The SCG3 protein is involved in the histamine metabolism [10].
ADCY9	Adenylate cyclase 9	16p13.3	Adenylate cyclase is a membrane bound enzyme that catalyses the formation of cyclic AMP from ATP. The type 9 adenylyl cyclase is a widely distributed adenylyl cyclase, and it is stimulated by beta-adrenergic receptor activation (https://www.ncbi.nlm.nih.gov/gene/115).
HRH4	Histamine receptor H4	18q11.2	The effects of the inflammatory mediator histamine are realized through its interaction with histamine receptors, including HRH4. The interaction of histamine with HRH1, HRH2, HRH3, and HRH4 receptors leads to their activation in the organism and increased production of cytokines [10; 15].
